# Litchi procyanidins inhibit colon cancer proliferation and metastasis by triggering gut-lung axis immunotherapy

**DOI:** 10.1038/s41419-022-05482-5

**Published:** 2023-02-11

**Authors:** Yuan Yao, Suya Feng, Xuejiao Li, Taohua Liu, Shengying Ye, Long Ma, Shuli Man

**Affiliations:** 1 State Key Laboratory of Food Nutrition and Safety, Key Laboratory of Industrial Microbiology, Ministry of Education, Tianjin Key Laboratory of Industry Microbiology, National and Local United Engineering Lab of Metabolic Control Fermentation Technology, China International Science and Technology Cooperation Base of Food Nutrition/Safety and Medicinal Chemistry, College of Biotechnology, Tianjin University of Science & Technology, Tianjin, 300457 China; 2 Henan Key Laboratory of Rare Diseases, Endocrinology and Metabolism Center, The First Affiliated Hospital, and College of Clinical Medicine of Henan University of Science and Technology, Luoyang, 471003 China; 3 Department of Pharmacy, The 983th Hospital of the Joint Logistics Support Force of the Chinese People’s Liberation Army, Tianjin, 300142 China

**Keywords:** Tumour immunology, Immunotherapy

## Abstract

*Litchi chinensis* seed, as a valuable by-product of the subtropical fruit litchi (*Litchi chinensis* Sonn.), has been confirmed to be rich in procyanidins (LPC). The anticarcinogenic properties of procyanidins has been primarily attributed to their antioxidant and anti-inflammatory activities. However, there is a comparative paucity of information on if and how LPC inhibits colon cancer. Here, LPC significantly inhibited CT26 colon cancer cells proliferation and metastasis in vivo and in vitro. In CT26 lung metastatic mice, the anti-metastatic effect of LPC relied on its regulation of gut microbiota such as increase of *Lachnospiraceae* UCG-006, *Ruminococcus*, and their metabolites such as acetic acid, propionic acid and butyric acid. In addition, LPC significantly inhibited CT26 colon cancer cells metastasis through increasing CD8^+^ cytotoxic T lymphocytes infiltration and decreasing the number of macrophages. Antibiotics treatment demonstrated that the therapeutic effect of LPC depended on the gut microbiota, which regulated T cells immune response. Taken together, LPC had strong inhibitory effects on colon cancer pulmonary metastasis by triggering gut-lung axis to influence the T cells immune response. Our research provides a novel finding for the utilization of procyanidins in the future, that is, supplementing more fruits and vegetables rich in procyanidins is beneficial to the treatment of colon cancer, or it can be used as an adjuvant drug in clinical anti-tumor immunotherapy.

## Introduction

Colorectal cancer (CRC) is the third most common malignancy and the second leading cause of cancer-related mortality in the world [1]. Metastasis is a primary driver of CRC-related mortality, with the liver and lungs representing the most frequently involved organs [2, 3]. The tumor microenvironment (TME) in CRC plays a key role in disease progression, therapy response, and overall survival. TME contains a heterogeneous cell population, such as endothelial, stromal, and immune cells, which secrete soluble signals (cytokines, chemokines, or growth factors), interact with tumor cells, and generate a favorable or unfavorable microenvironment to adjust tumor growth and metastasis [4]. For example, tumor-infiltrating lymphocytes prolong survival rate of CRC patients [5]. CD4^+^ T cells are key mediators of the adaptive immune system, secreting cytokines to modulate other immune cells in response to cancer [5]. CD8^+^ cytotoxic T lymphocytes (CTLs) participate in adaptive immune response and play crucial roles in antitumor immunity [6]. Meanwhile, CTLs is exhausted to promote immune escape [7]. Myeloid-derived suppressor cells or tumor-associated macrophages (TAMs) are associated with poor prognosis [8, 9].

The cause of CRC is multifactorial including environmental factors, diet, genetic predisposition, and epigenetic alterations in the colonic epithelium [10]. Meanwhile, microbes are involved in approximately 20% of cancer, especially for CRC [11]. For example, SCFAs-producing bacteria such as *Roseburia*, *Lachnospiraceae* and *Ruminococcus* decreased in colorectal cancer, which is disadvantageous to cancer immunotherapy [12, 13]. Thus, the strategies for modulating gut microbiota have been proposed to treat cancer patients.

Litchi (*Litchi chinensis* Sonn.), commonly known as a fruit, is cultivated in semitropical areas for its palatable pulp. Its seeds are rich in various procyanidins, such as procyanidin A, procyanidin D, cinnamtannin B1, cinnamtannin B2, etc. [14, 15]. Procyanidins can induce cellular apoptosis and exhibit anti-angiogenesis, anti-metastasis, anti-inflammatory and antioxidant activity [16]. Meanwhile, procyanidins are regarded as a prebiotic which can be degraded by gut microbiota and metabolized into various phenolic compounds. These metabolites contribute to the benefits of human and treat ulcerative colitis [17], obesity [18], atherosclerosis [19], and diabetic [20]. However, it is unclear whether procyanidins act as an immunomodulator by regulating gut microbiota in the treatment of colon cancer. Therefore, we set up colon cancer and colon cancer metastatic models to evaluate the effect of litchi seed procyanidins (LPC).

## Results

### LPC inhibits CT26 colon cancer in vitro and in vivo

**Fig. 1:**
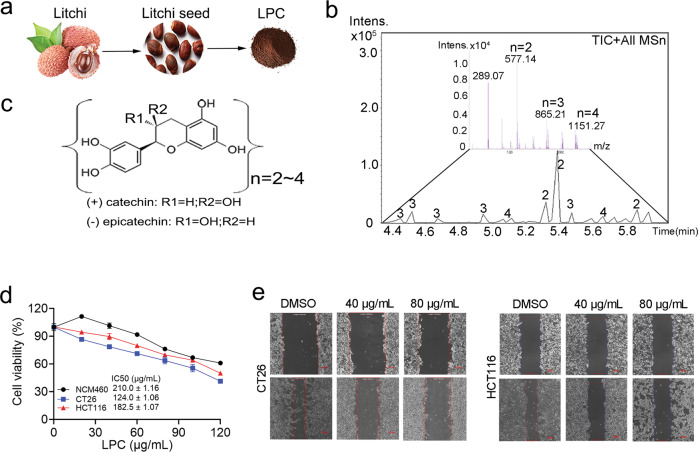
LPC inhibits the proliferation and migration of CT26 colon cancer cells. **a** Source of LPC. **b** Identification of LPC by UPLC-Q/TOF-MS. **c** Inferred composition of LPC. **d** Cell viability was measured by MTT assay (*n* = 4). **e** Cell migration was measured by wound healing assay. Data were presented as means ± SEM.

LPC was extracted from *litchi chinensis* seed (Fig. 1a). The chemical composition of LPC was identified by UPLC-Q/TOF-MS, which contained four kinds of procyanidin oligomers with 2~4 degree of polymerization (Fig. 1b, c). To reveal the potential anti-colon cancer mechanisms of LPC, tumor inhibition was evaluated in vitro and in vivo. As a result, LPC concentration-dependently inhibited the cell viability of CT26 and HCT116 colon cancer cells (Fig. 1d). Interestingly, the killing effect of LPC on normal colon cells (NCM460) was weaker than that on CT26 and HCT116 colon cancer cells (Fig. 1d). The wound healing assay demonstrated that LPC significantly inhibited CT26 and HCT116 cells migration (Fig. 1e). In addition, LPC treatment significantly decreased the tumor volume and tumor weight compared with M group, (Supplementary Fig. 5d, e). However, it did not decrease the body weight of mice (Supplementary Fig. 5c). Taken together, LPC were safe and significantly inhibited CT26 cell proliferation and tumor growth in vitro and in vivo.

### LPC inhibits pulmonary metastasis of CT26 colon cancer

**Fig. 2:**
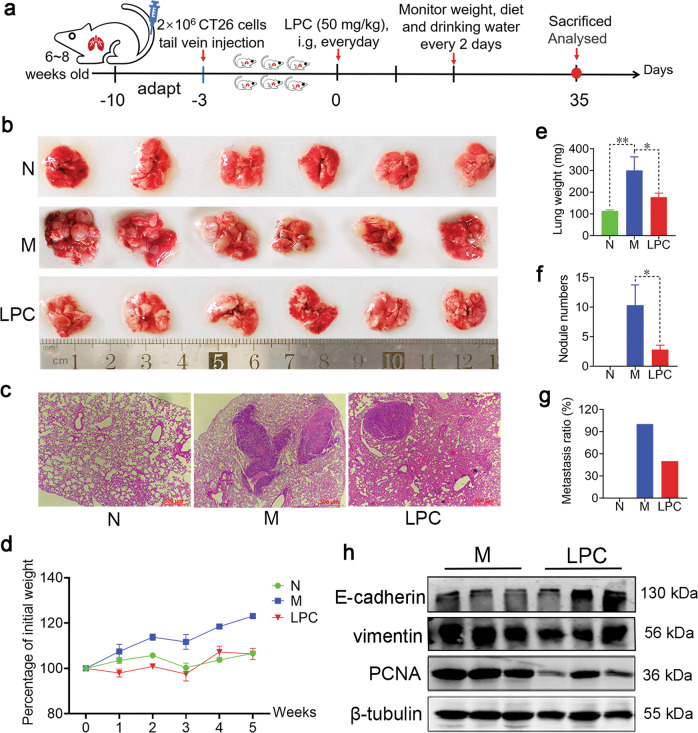
LPC inhibits pulmonary metastasis of CT26 colon cancer. **a** Schematic view of the experimental procedures of CT26 pulmonary metastatic mouse model. **b**, **c** Image and corresponding H&E staining of lung tissue. **d** Percentage change of body weight. **e**–**g** Lung weight, the number of lung tumor nodule, and metastasis rate (*n* = 6 mice). **h** Protein expression of PCNA, vimentin and E-cadherin in lungs (*n* = 3 mice). Data were presented as mean ± SEM, *p < 0.05, **p < 0.01.

To investigate whether LPC inhibit metastasis of colon cancer, we successfully established a pulmonary metastatic mouse model via injecting CT26 cells in tail vein [21] (Fig. 2a). Within 5 weeks, the whole M mice developed pulmonary metastasis as opposed to 3 out of 6 in LPC group, suggesting that LPC inhibit the pulmonary metastasis of CT26 colon cancer (Fig. 2b). Besides, LPC treatment dose-dependently slowed the increase of lung weight (Supplementary Fig. 1a) and decreased the metastasis rate and the number of tumor nodules compared with M group (Fig. 2e–g and Supplementary Fig. 1a–d). H&E staining displayed the smaller and less lung metastatic lesions in LPC group compared with that in the M group (Fig. 2c). Meanwhile, there were no significant changes in terms of body weight, food intake, or water intake among three groups (Fig. 2d and Supplementary Fig. 1e–g). Furthermore, LPC treatment significantly decreased the protein expression of PCNA and vimentin, and increased the protein expression of E-cadherin in lungs compared with that in the M group (Fig. 2h). Together, these data demonstrated that LPC inhibited the proliferation and metastasis of CT26 cells in lungs.

### LPC activates T cell immunity to inhibit the colonization of CT26 cells in lungs

**Fig. 3:**
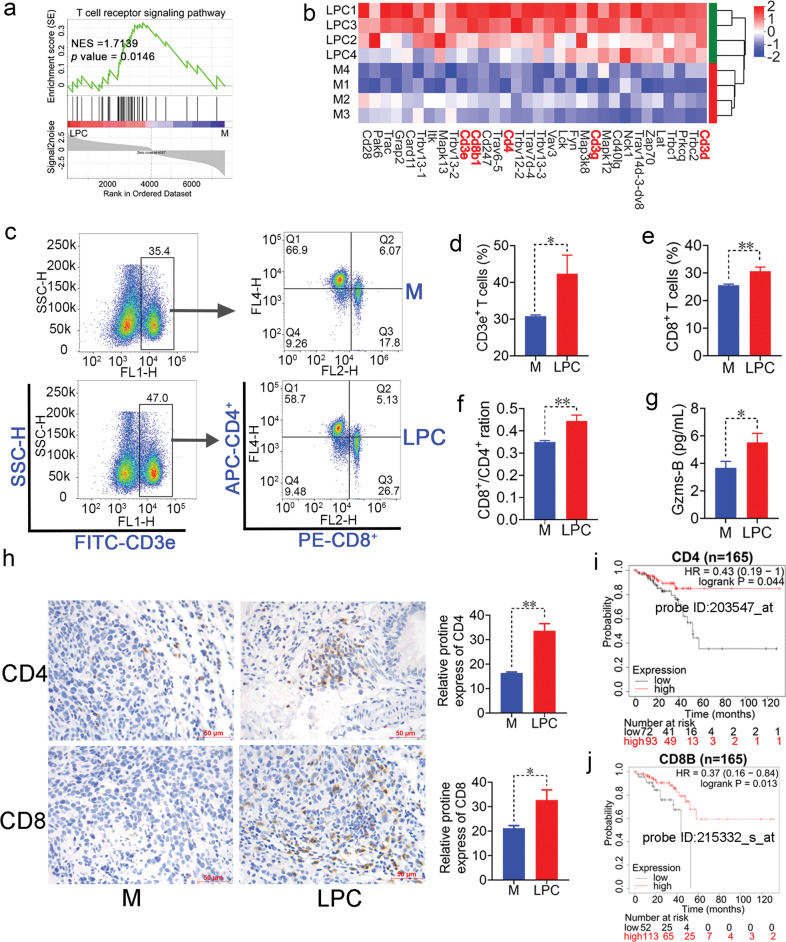
LPC regulates T cell immunity in pulmonary metastasic mice. **a** GSEA based on T cell receptor signaling pathway. **b** Heatmap plot of gene set involved in T cell receptor signaling pathway. **c** T cells were detected by flow cytometry. **d**–**f** The relative amount of CD3e^+^ T, CD8^+^ T, and the ratio of CD8^+^/CD4^+^ (*n* = 3 mice). **g** The serum level of Gzms-B (*n* = 5 mice). **h** Representative IHC profile of CD4 and CD8 in lungs (*n* = 3 mice). 400× magnification, scale bar = 50 µm. **i**, **j** Kaplan-Meier curves of the relationship between gene expression of CD4, CD8B and survival probability in colorectal cancer patients. Data were presented as mean ± SEM, *p < 0.05, **p < 0.01.

The integration of different genes hitting into particular signaling pathways and cellular functions is key to understand how LPC inhibits colon metastasis. As a result, the gene structure was notably changed after LPC administration (Supplementary Fig. 2a, b). There were 4060 upregulated genes and 3552 downregulated genes in LPC group (Supplementary Fig. 2c). KEGG functional annotation analysis showed that these differential genes were enriched in immune system under Organismal Systems classification (Supplementary Fig. 2d), such as T cell receptor signaling pathway, IL-17 signaling pathway, leukocyte transendothelial migration, etc. (Fig. 3a and Supplementary Fig. 2e, i–j). Among these pathways, LPC treatment increased gene expression of Cd3e, Cd3d, Cd3g, Cd4, and Cd8b involved in T cell receptor signaling pathway (Fig. 3b). Interestingly, High expression of CD3E, CD3D, CD3G, CD4, CD8B were closely related to the higher survival probability (Fig. 3i–j and Supplementary Fig. 2f–h). Flow cytometry analysis indicated that LPC treatment significantly up-regulated the peripheral number of CD8^+^ cytotoxic T cells and increased the ratio of CD8^+^/CD4^+^ (Fig. 3c–f). Immunohistochemical results showed that LPC treatment upregulated protein expression of CD4 and CD8 in lung microenvironment (Fig. 3h). Meanwhile, the increase of serum Gzms-B, an indicator of activated cytotoxic T-cell, supported the above inference that LPC increased the activity of cytotoxic T-cell (Fig. 3g). In addition, the serum level of IL-17A, an inflammatory factor that promoted the growth and metastasis of colon cancer [22], was also significantly decreased (Supplementary Fig. 2k) in LPC group. Overall, these data suggested that LPC inhibit lung metastasis of CT26 cells by modulating the T cell immune response.

### LPC inhibits TAMs and inflammatory factors accumulation

**Fig. 4:**
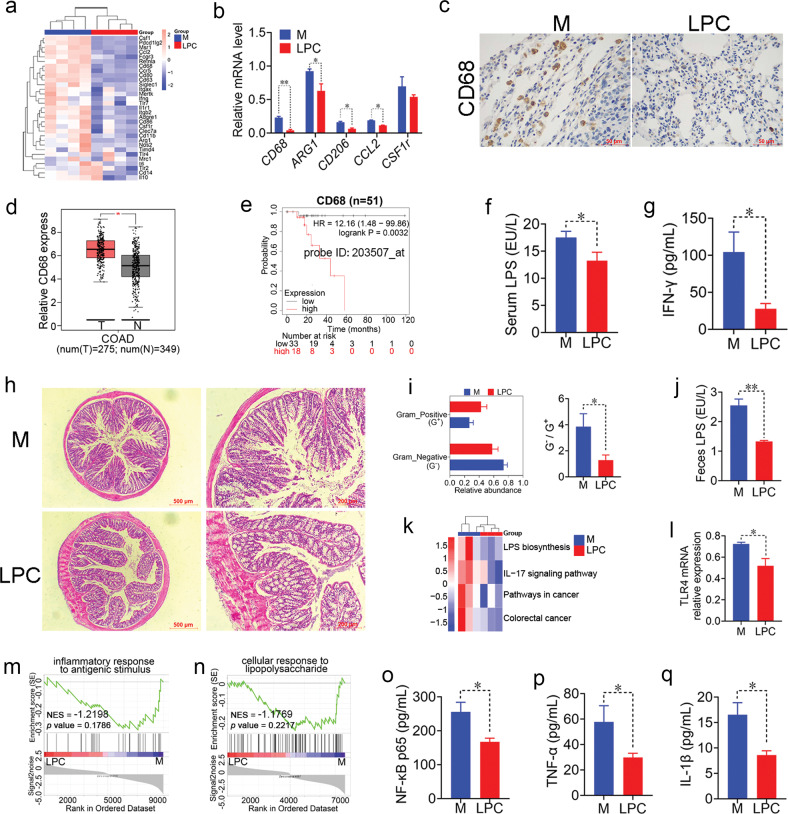
LPC inhibits TAMs and inflammatory factor accumulation in pulmonary metastatic mice. **a** Heat map representing macrophage related genes profile. **b** The gene expression of macrophage markers such as CD68, ARG1, CD206, CCL2 and CSF1R in lungs (*n* = 3 mice). **c** The protein expression of CD68 in lungs. **d** Expression of CD68 gene profile in colon cancer patients. **e** Kaplan-Meier curves of the relationship between gene expression of CD68 and survival probability in advanced colorectal cancer patients. **f**, **g** The serum levels of LPS and IFN-γ (*n* = 3 and 6 mice). **h** Histomorphology of the colon. **i** The proportion of G^−^ and G^+^ microbes within microbiomes of stools, as predicted by BugBase (*n* = 4 mice). **j** The level of fecal LPS (*n* = 5 mice). **k** Functional profiling of microbiome throughout disease progression. **l** mRNA expression of TLR4 in colon tissue (*n* = 3 mice). **m** GSEA based on inflammatory response to antigen stimulates. **n** GSEA based on cellular response to lipopolysaccharide. **o**–**q** The serum levels of NF-κB p65, TNF-α and IL-1β (*n* = 4 mice). Data were presented as mean ± SEM, *p < 0.05, **p < 0.01.

TAMs as the predominant infiltrated immune cells in TME exhibit pro-tumoral angiogenesis, metastasis, and immunosuppression activity [23, 24]. In present study, transcriptome analysis indicated that macrophage related genes were significantly down-regulated in LPC group (Fig. 4a). Among these genes, the key markers of macrophage such as CD68, ARG1, CD206 and CCL2 were significantly inhibited in LPC group compared with that in M group, especial for the change of CD68 (Fig. 4b, c). Importantly, CD68 reached relatively high expression in advanced colorectal cancer patients (Supplementary Fig. 3) and had negative correlation with their survival probability (Fig. 4e). Meanwhile, LPC treatment significantly inhibited the activators of TAMs such as LPS and IFN-γ [25] in serum (Fig. 4f, g). These findings demonstrated that LPC inhibited the activity and the number of macrophages in TME.

**Fig. 5:**
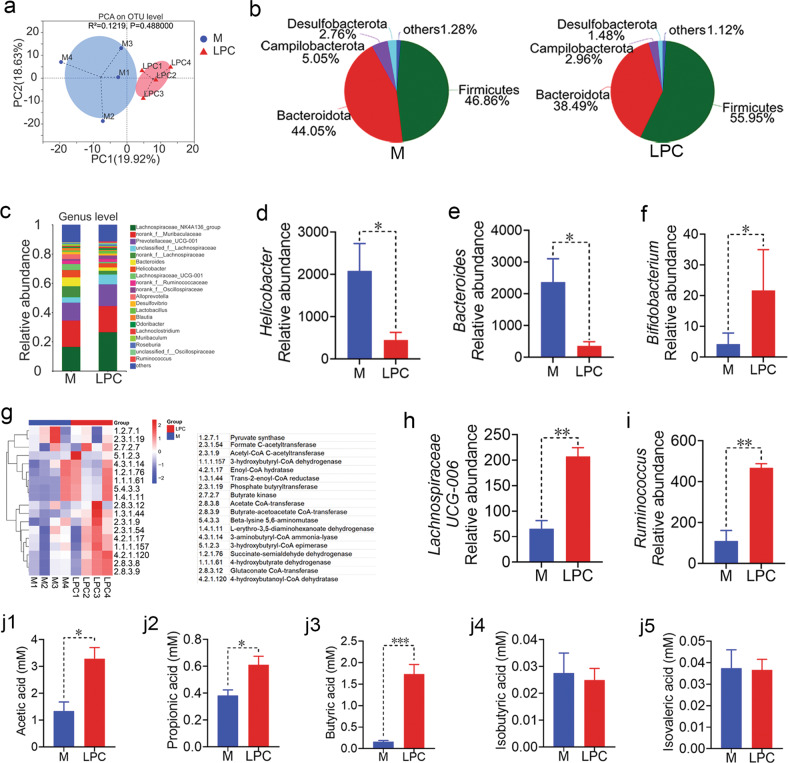
LPC regulates gut microbiota and their metabolites in pulmonary metastatic mice. **a** PCA plot with Bray-Curtis distance analysis at OTU level. **b** Abundance of major bacterial phyla. **c** Abundance of major bacterial genus. **d**–**f** Relative abundance of *Helicobacter*, *Bacteroides* and *Bifidobacterium* (*n* = 4 mice). **g** The gene copies of butyric acid synthesis related enzymes in each group (*n* = 4 mice). **h**, **i** Relative abundance of *Lachnospiraceae UCG-006* and *Ruminococcus* (*n* = 4 mice). The levels of fecal SCFAs such as **j1** acetic acid, **j2** propionic acid, **j3** butyric acid, **j4** isobutyric acid and **j5** isovaleric acid (*n* = 4 mice). Data were presented as mean ± SEM, *p < 0.05, **p < 0.01, ***p < 0.001.

Furthermore, gut microbiota is closely related to the macrophages [26]. LPS is the outer membrane of gram‐negative (G^−^) microbes, such as *Escherichia coli* and *Bacteroides*, which stimulate macrophages and induce an inflammatory response [27]. At the organism-level, the enriched proportion of G^−^ microbes and *Bacteroides* were decreased after LPC treatment (Figs. 4i, 5b). In addition, according to the PICRUSt2 functional prediction analysis of gut microbiota, LPS synthesis pathway was enriched in M group (Fig. 4k). LPC treatment significantly decreased the level of LPS in colons (Fig. 4j).

If the integrity of intestinal endothelial barrier is destroyed, the intestinal LPS would invade the body. Subsequently, LPS binding to TLR4 transmits inflammatory signals in the intestine to promote the progression of CRC [28]. In this research, histopathological evaluation of the colon revealed that LPC treatment protected the integrity of intestinal endothelial barrier against the invasion of pathogenic microorganisms (Fig. 4h). Intestinal epithelial TLR4 gene was significantly decreased after LPC treatment (Fig. 4l). The gene set of CD68 correlative ‘inflammatory response to antigen stimulus’ and ‘cellular response to lipopolysaccharide’ were relatively enriched in M group compared with that in the LPC group (Fig. 4m, n). The inflammatory factors such as NF-κB p65, TNF-α and IL-1β were significantly inhibited in LPC-treated serum (Fig. 4o–q). These results indicated that LPC inhibited G^-^ bacteria producing LPS, and suppressed inflammatory factors and TMAs accumulation in CT26 pulmonary metastatic mice.

### LPC regulates gut microbiota and increases SCFAs

To elucidate the impact of LPC on the gut microbiome, the microbiota composition was analyzed in CT26 pulmonary metastatic mice. Rarefaction-curve analysis displayed 97% similarity in terms of OTUs. The rarefaction curve in each group was almost saturated, which indicated that sufficient sequencing data were obtained to reflect nearly all microbial diversity in each group (Supplementary Fig. 4a). Alteration in the make-up of microbial community was observed between M and LPC mice (Fig. 5a). While the richness and evenness of the gut microbiota was not significantly changed (Supplementary Fig. 4b, c). At the phylum level, the relative abundance of *Firmicutes* was increased, while *Bacteroidota*, *Campilobacterota* and *Desulfobacterotain* were decreased after LPC treatment (Fig. 5b). At the genus level, the relative abundance of *Helicobacter* and *Bacteroides* were decreased (Fig. 5d, e), while probiotics such as *Bifidobacterium* and *Lactobacillus* were increased after LPC treatment (Fig. 5f and Supplementary Fig. 4d).

SCFAs as crucial metabolites serve as an energy source, maintain intestinal epithelial integrity, and mediate the contribution of the microbiota to cancer immunity. To explore the biosynthetic mechanisms of SCFAs in the gut, PICRUSt2 function prediction was employed to analyze the change of enzymes participating in SCFAs biosynthesis. As a result, LPC treatment up-regulated the number of enzymes responsible for final butyric acid production (Fig. 5g). The concentration of fecal acetic acid, propionic acid and butyric acid was also significantly increased (Fig. 5j1-j3), along with their positively SCFAs-producing bacteria such as *Lachnospiraceae UCG-006*, *Ruminococcu*, *Lachnospiraceae NK4A136* and *Roseburia* in LPC group (Fig. 5h, i and Supplementary Fig. 4e, f). Pearson correlation analysis indicated that there was a significant positive correlation between CD8 cells and *Lachnospiraceae UCG-006*, *Ruminococcu*, acetic acid and butyric acid (Supplementary Fig. 4g–i). Altogether, these findings suggested that the immune response of LPC against pulmonary metastasis may be related to its regulation of gut microbiota and their metabolites like SCFAs.

### LPC-triggered anti-colon cancer T cell immune response depends on gut microbiota

**Fig. 6:**
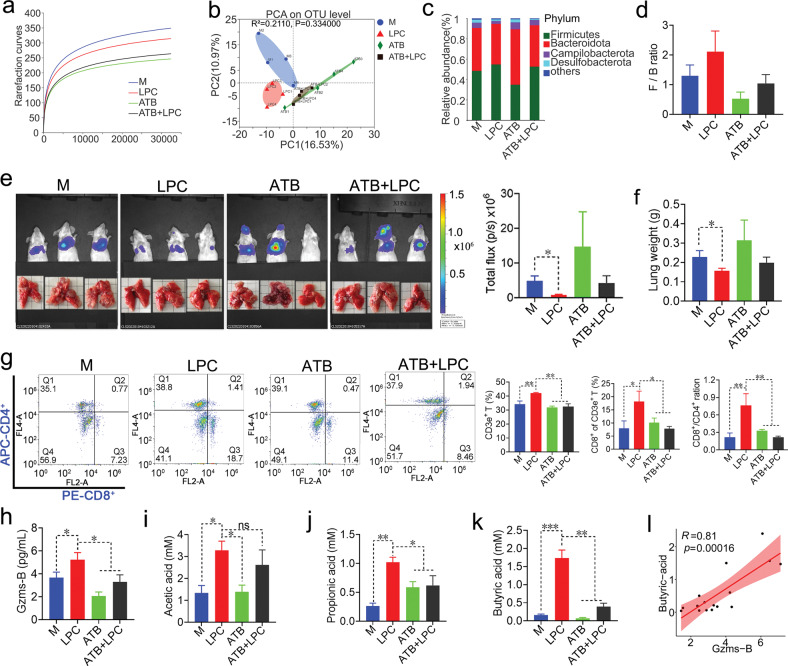
LPC-triggered anti-colon cancer T cell immune response depends on gut microbiota. **a** Rarefaction curves at the OTU level. **b** PCA plot with Bray-Curtis distance analysis at OTU level. **c** Abundance of major bacterial phyla. **d** Ratio of F/B (*n* = 3 mice). **e** Pulmonary metastasis of CT26 luciferase cells by in vivo imaging of luminescence (*n* = 3 mice). **f** Average lung weight at the end of experiment (*n* = 6 mice). **g** Flow cytometry analysis of CD3e^+^ T and CD8^+^ T in spleen (*n* = 3 mice). **h** The content of Gzms-B in serum (*n* = 5 mice). **i**–**k** The level of acetic acid, propionic acid and butyric acid in colons (*n* = 5 mice). **l** Pearson correlation analysis between Gzms-B and butyric acid. R was correlation coefficient. Data were presented as mean ± SEM, ns means no significance, *p < 0.05, **p < 0.01, ***p < 0.001.

In order to confirm that LPC inhibits CT26 pulmonary metastasis dependently on gut microbiota, we used broad-spectrum antibiotic rifaximin (ATB) to destroy the composition of gut microbiota and evaluate antitumor effect of LPC and ATB (Supplementary Fig. 5a). As expected, the gut microbiota structure was changed after ATB treatment (Fig. 6a, b). ATB treatment weakened the anti-tumor (Supplementary Fig. 5b, d, e) and anti-metastatic effects (Fig. 6e, f) of LPC, but didn’t reduce the body weight of mice (Supplementary Fig. 5c). Meanwhile, the relative abundance of SCFAs-producing bacteria, such as *Lachnospiraceae UCG-006*, *Ruminococcus* and *Roseburia* was significantly decreased in and ATB + LPC groups compared with that in the LPC group (Supplementary Fig. 5f–h). The levels of CD3e^+^ T, CD8^+^ T, Gzms-B, butyric acid and propionic acid were also significantly decreased in ATB + LPC group compared with that in LPC group (Fig. 6g, h and Fig. 6j, k). Furthermore, there was a strong correlation between Gzms-B and butyric acid (R = 0.81), in contrast to acetic acid (R = 0.46) and propionic acid (R = 0.52) (Fig. 6l and Supplementary Fig. 5j, k). These results indicated that LPC-triggered anti-colon cancer T cell immune response depend on gut microbiota and SCFAs, especially for butyric acid.

## Discussion

Procyanidins, a kind of dietary polyphenol compounds rich in lichi seed, tea leaves, cacao, grapes and apples, display various physiological activities such as anti-cancer [16], anti-diabetes [29], cardiovascular protection [30] and immune-regulation [31]. LPC contains three procyanidin oligomers with polymerization degree of 2~4, mainly type A procyanidins. In this study, LPC significantly inhibited CT26 colon cancer growth and its pulmonary metastasis in vitro and in vivo. Lung is regarded as the second most common metastatic site in CRC [21]. As we known, cell adhesion molecules play an important role in the metastases, which not only mediate the adhesion of tumor cells to tumor cells, vascular endothelial cells, lymphocytes, and extracellular matrix, but also mediate the migration of tumor cells across endothelial cells [32]. In this research, LPC treatment significantly increased protein expression of E-cadherin, which reduced the epithelial-mesenchymal transition of tumor cells [32, 33], and reduced the protein expression of vimentin in lungs that participated in the adhesion, migration, invasion and cell signal transduction of tumor cells, tumor related endothelial cells and macrophages [34].

The TME of CRC plays a key role in disease progression, therapy response, and overall survival. There are a diverse array of immune cells including macrophages, lymphocytes and dendritic cells in pulmonary microenvironment, which are crucial for defensing against airborne pathogens, toxins and inflammatory substances, and maintaining the balance of the body homeostasis. In contrast, some of the immune cells contribute to tumor engraftment and metastasis [4]. Here, transcriptome analysis as a general view was used to understand how LPC inhibited tumor growth and metastasis through immune system. As a result, T cell receptor signaling pathway and leukocyte transendothelial migration were significantly enriched in LPC group. This indicated when the organism detected CT26 cells, the immune cells passed through the capillary wall and gather at the lesions to eliminate heterologous substances [35]. Furthermore, the ratio of CD8^+^/CD4^+^ and the content of Gzms-B significantly increased in LPC group compared with that in the M group, demonstrating that LPC could activate cytotoxic T cells to kill CT26 cells.

TAMs as one of the main tumor-infiltrating immune cells produce pro-inflammatory cytokines, such as NF-κB p65, IL-1β and TNF-α and promote the development of CRC [36]. As previous reported, procyanidin A1 as the main compound in LPC alleviated LPS-induced macrophages inflammatory response through NF-κB pathway [37]. In this experiment, LPC treatment decreased the number of TAMs in TME, inhibited their activators like LPS and IFN-γ [27, 38], and reduced the levels of inflammatory factors, such as NF-κB p65, TNF-α, and IL-1β. As previous reported, NF-κB p65, IL-1β and TNF-α, as key signaling molecules, induced the production of IL-17 [39], which promoted the proliferation and metastasis of colon cancer [40]. Transcriptome analysis indicated that LPC could significantly inhibit IL-17 signaling pathway. The serum level of IL-17A also decreased after LPC treatment.

Polyphenols are the most common plant-derived bioactive components in our diet. Most of the polyphenols intake remained unabsorbed in the small intestine [41]. Unabsorbed polyphenols may be accumulated in the gut and metabolized by the gut microbiota into a series of low-molecular-weight polyphenolic metabolites that can be readily absorbed and confer health benefits [41]. Meanwhile, oligomers procyanidins were rarely (DP = 2~4) [42] or not (DP > 4) [43] absorbed into body. Most procyanidins reach the intestine and play prebiotic roles against disease. Here, it was found that the intestinal epithelial cells of LPC group were more complete than that of M group, implying that LPC inhibited pathogenic substances crossing over the intestinal barrier to promote CT26 development. LPC also inhibited the gene expression of TLR4 in colonic epithelial cells, which received LPS and mediated inflammatory signals [28]. Furthermore, PCA analysis indicated that there were significant differences in terms of bacterial community structure between LPC and M groups. *Helicobacter* can activate pro-inflammatory Th17 signaling pathway with co-expression of IL-17 and IFN-γ [44]. *Bacteroidetes* can produce LPS, which induces tissue inflammation, acts on TLR4 and promotes tumorigenesis [45]. The relative abundance of *Helicobacter* and *Bacteroidetes* significantly declined after LPC treatment.

In addition, the number of SCFAs-producing bacteria such as *Lachnospiraceae NK4A136*, *Ruminococcu* and *Roseburia*, and the levels of fecal SCFAs were higher in LPC group than that in M group [46, 47]. The immune-regulative effect of LPC may depend on SCFAs that increase the number of CD8^+^ T cells [48], down-regulate the number of regulatory T cells to eliminate immunosuppression [49], and inhibit inflammation in organs located outside of gut [50, 51]. To further verify the necessity of gut microbiota on the antitumor immunity triggered by LPC, we used a broad-spectrum antibiotic to disturb the gut microbiota. As expected, the anti-tumor effect of LPC was weakened by ATB. Our study demonstrated that the gut-lung axis played an important role in anti-tumor effect of LPC.

In summary, we developed a potential antitumor effect of LPC in colon cancer therapy. LPC significantly inhibited the proliferation and metastasis of colon cancer by increasing the number of CD8^+^ T cells and decreasing the number of macrophages in TME. The gut-lung axis is a bridge for LPC to regulate T cell immune response to inhibit the proliferation and metastasis of colon cancer, which underscoring LPC promoted the generation of SCFAs which played the key roles in induction of CD8^+^ T cell infiltration in TME. Therefore, our research provides a novel, conceptually novel paradigm for colon-cancer immunotherapy for the utilization of procyanidins in the future, that is, supplementing more fruits and vegetables rich in procyanidins is beneficial to the treatment of colon cancer, or therapeutically, it can be used as an adjuvant drug in clinical anti-tumor immunotherapy.

## Materials and methods

### Materials

LPC with a purity of more than 95% was extracted from litchi seed by MuFan Biotech Co., Ltd (Henan, China). The identity of the LPC was characterized by UPLC-Q/TOF-MS (Bruker, Germany). Cell lines NCM460, CT26 and HCT116 are purchased from BeNa Culture Collection (Beijin, China). The cells were maintained in RPMI 1640 supplemented plus 10% FBS, and 1% penicillin‐streptomycin (Solarbio, China) at 37 °C in a humidified in humidified air containing 5% CO_2_. CT26 cells were transduced with retrovirus expressing the triple-fusion reporter gene encoding herpes simplex virus thymidine kinase 1, GFP and firefly luciferase for bioluminescence imaging for lung metastasis in vivo [21].

### MTT assay

Effects of LPC on viability of NCM460, CT26, HCT116 cells were measured by a colorimetric assay using MTT assay kit (Solarbio, China). NCM460, CT26, HCT116 cells were seeded at a density of 1 × 10^3^/well in a complete growth medium in 96-well plates for 4 duplicates. The cells were incubated with the test compounds for 24 h before the MTT assay. Then, MTT (0.5 mg/mL) was added to each single well with a further incubation for 4 h. Finally, the formazan was dissolved with 100 μL of DMSO and then analyzed in a multiwall plate reader at 570 nm (BioTek Instruments, USA).

### Wound healing assay

CT26 and HCT116 cells at 1 × 10^6^/ well in 6 well-plates until cells were confluent or nearly (>90%) confluent. Cell monolayers were scratched by using a 10-µl pipette tip, and then rinsed three times with 1 × PBS to remove cell debris. Then treating with different concentrations of LPC. Cell migration in the wound area was observed by phase contrast microscopy at 0 and 48 h and digitally photographed.

### Animal studies

The 8 weeks old BALB/c female mice were purchased from Skbex Botechnology (HeNan, China). The animals were kept at room temperature and humidity-controlled room with 12 h light/dark cycle. All the mice adapted to the living environment for one week. For subcutaneous or tail vein injections 2 × 10^6^ CT26 cells were suspended in 200 µL of cold PBS. Mouse were randomly divided into five groups: including normal (N) and model (M) group treated with PBS, LPC treatment (25, 50, 100 mg/kg, ig.), antibiotic rifaximin (ATB, 250 mg/L in drinking water), and ATB + LPC (50 mg/kg) treatment (six in each group). Tumor volume was recorded every three days until the end point according to the formula: volume (mm^3^) = 0.52 × L × W^^2^, where L is the length and W is the width of the tumor (in millimeters).

In vivo bioluminescence (BLI) was performed at fluorescent-labeled CT26 cells into BALB/c mice. After the mice were anesthetized with 5% pentobarbital sodium and intraperitoneally injected with D-luciferin (150 mg/kg, ab143654, Abcam, USA) 20 min. They were transferred to the imaging chamber. BLI in terms of photon emission per second was recorded from each mouse by using intelligent visualization software (IVIS) imaging system (PerkinElmer Inc., Waltham, MA) at the optimal imaging time. The BLI data were quantitated by using IVIS software.

### ELISA assay

Serum levels of LPS (RXJ202425M), Gzms-B (RX202790M), IL-1β (RX203063M), IL-17A (RX203066M), NF-κB p65 (RX201879M), TNF-α (RX202412M), and IFN-γ (RX203097M) were measured by kits according to the manufacturer’s instructions, purchased from Ruixin Biotechnology Co., Ltd. (Quanzhou, China).

### Histology examination

In short, samples were fixed in 4% paraformaldehyde, and embedded in paraffin for hematoxylin-eosin staining (H&E) or immunohistochemical analysis. Samples were incubated with CD4 (1:50, GB13064-1, Servicebio), CD8 (1:200, GB11068-1, Servicebio), and CD68 (1:200, GB14043, Servicebio) at 4 °C overnight and secondary antibody labeled with horseradish peroxidase at room temperature for 2 h. Finally, samples were counterstained with hematoxylin and visualized with 3,3’-diaminobenzidine (DAB) tetrahydrochloride for 10 min. All the sections were examined and the pictures were taken by digital camera (Leica, ICC50 W) and protein expressions were quantified by Image-Pro Plus 6.0.

### Flow cytometry

In brief, at the endpoint of the experiment, spleens or tumors were harvested for flow cytometry analysis. Subsequently, single-cell suspensions were stained with the following antibodies: FITC anti-mouse CD3 (F21003A01), APC anti-mouse CD4 (F2100403), and PE anti-mouse CD8 (F21008A02) were purchased from MULTISCIENCES (Hangzhou, China). The cell suspension was incubated for 15 min at RT in the dark, and analyzed by flow cytometry (Accuri C6, USA) within 1 h. Data were analyzed using FlowJo software (V.10.4, FlowJo).

### Western blot

The harvested lungs (including lung metastatic foci and paracancerous tissue) were lysed with a RIPA buffer supplemented with PMSF (Solarbio, China) and protease inhibitors (MCE, USA). The protein concentrations were determined by using G-250 kit (Solarbio, China). Equal amounts of proteins were separated on SDS-PAGE and transferred to PVDF membranes (Millipore, Billerica, MA). The primary antibodies included β-tubulin (1:5000, 250175, ZEN BIO, China), E-cadherin (1:500, PB9561, BOSTER, China), vimentin (1:500, PB9359, BOSTER, China) and PCNA (1:2000, 10205-2-AP, Proteintech, China). Then protein band was detected by Odyssey Infrared Imaging System (LI-COR Biotechnology, USA). The relative level of each protein was quantified by using Lane 1D image analysis software (Sagecreation, China).

### Reverse transcription PCR

In brief, total RNA was extracted by using Trizol reagent (Solarbio, China) from lungs (including lung metastatic foci and paracancerous tissue). RNA quantity was determined by using BioSpectrometre (Eppendorf, Germany), and transcribed to cDNA using reverse transcription reagents (Solarbio, China). Relative mRNA expression was detected by RT-PCR using agarose electrophoresis. Amplification of the sequence of interest was normalized with the reference endogenous gene *GAPDH*. The primer of target genes displayed in Supplementary Table 1.

### Transcriptome analysis

In brief, Total RNA was extracted from lungs (including lung metastatic foci and paracancerous tissue) using TRIzol® Reagent according the manufacturer’s instructions (Invitrogen) and genomic DNA was removed using DNase I (TaKara). RNA-seq transcriptome librariy was prepared following TruSeqTM RNA sample preparation Kit from Illumina (San Diego, CA) using 1 μg of total RNA. Shortly, mRNA was isolated according to polyA selection method by oligo (dT) beads and then fragmented. Double-stranded cDNA was synthesized using a SuperScript double-stranded cDNA synthesis kit (Invitrogen, CA) with random hexamer primers (Illumina). Then the synthesized cDNA was subjected to end-repair, phosphorylation and ‘A’ base addition according to Illumina’s library construction protocol. Libraries were size selected for cDNA target fragments of 300 bp on 2% Low Range Ultra Agarose followed by PCR amplified using Phusion DNA polymerase (NEB) for 15 PCR cycles. After quantified by TBS380, paired-end RNA-seq sequencing library was sequenced with the Illumina HiSeq xten/NovaSeq 6000 instrument in Shanghai Majorbio Biopharm Technology Co. Ltd (Shanghai, China).

### 16 S rRNA gene sequencing of the gut microbiota

Microbial community genomic DNA was extracted from M, LPC, ATB and ATB + LPC groups using the E.Z.N.A.® soil DNA Kit (Omega Bio-tek, Norcross, GA, U.S.) according to manufacturer’s instructions. The detailed process refers to previous studies [14].

### Fecal SCFAs analysis

Gas chromatography was used to detect the content of SCFAs in feces. In brief, 50 mg of feces was suspended in 0.5 mL of Mili Q water and homogenized for 20 s under 6500 rpm for three times with an interval of 10 s by HF-24 homogenizer (Hefan Instrument, China). The standard mixture of SCFAs included acetic acid, propionic acid, butyric acid, isobutyric acid, isovaleric acid and isohexanoic acid (Aladdin, China). The concentration of SCFAs was detected according to the standard curve by internal standard method. The detailed process refers to previous studies [14].

### Bioinformatics analysis

The expression data of CD68 in colorectal cancer patients at various stages were obtained from GEPIA (http://gepia.cancer-pku.cn/index.html). The survival curves of specific genes were drawn from Kaplan-Meier Plotter (https://kmplot.com/analysis/).

### Statistical analyses

The SPSS 21.0 version software was used for statistical analysis. All experiments were performed at least three times. The data were expressed as means ± SEM. One-way analysis of variance (one way-ANOVA) with LSD’s multiple comparison test was used to assess differences between more than two groups. No statistical methods were used to predetermine the sample size. Mice were randomly allocated to experimental groups. No blinding method was used for injection. There was no animal exclusion criteria. Survival analysis was performed using the Kaplan-Meier estimates and the log-rank test. ns means no significance, *p < 0.05, **p < 0.01, ***p < 0.001.

## Supplementary information

Supplementary Information

Original Data File

Reproducibility checklist

## Data Availability

All datasets generated and analyzed during this study are included in this published article and its Supplementary Information files. Additional data are available from the corresponding author on reasonable request.
