# Supplementary material for: Litchi procyanidins inhibit colon cancer proliferation and metastasis by triggering gut-lung axis immunotherapy
Source: Cell Death Dis. 2023 Feb 11;14(2):109. doi: 10.1038/s41419-022-05482-5 (PMC9922286; doi:10.1038/s41419-022-05482-5)
Supplement: Supplementary file 1 — Supplementary Information [file 41419_2022_5482_MOESM1_ESM.doc]

**Supplementary Information**

**Litchi procyanidins inhibit colon cancer proliferation and metastasis by triggering gut-lung axis immunotherapy**

Yuan Yao^1 a^, Suya Feng^1 a^, Xuejiao Li^2 a^, Taohua Liu^1^, Shengying Ye ^iD 3^, Long Ma ^iD 1^, Shuli Man ^iD 1^

^1^ State Key Laboratory of Food Nutrition and Safety, Key Laboratory of Industrial Microbiology, Ministry of Education, Tianjin Key Laboratory of Industry Microbiology, National and Local United Engineering Lab of Metabolic Control Fermentation Technology, China International Science and Technology Cooperation Base of Food Nutrition/Safety and Medicinal Chemistry, College of Biotechnology, Tianjin University of Science & Technology, Tianjin, 300457, China

^2^ Henan Key Laboratory of Rare Diseases, Endocrinology and Metabolism Center, The First Affiliated Hospital, and College of Clinical Medicine of Henan University of Science and Technology, Luoyang, 471003, China

^3^ Department of Pharmacy, The 983th Hospital of the Joint Logistics Support Force of the Chinese People’s Liberation Army, Tianjin, 300142, China

Correspondence: shengyingye@163.com; malong@tust.edu.cn; msl@tust.edu.cn

^iD^ Shengying Ye: 0000-0001-8055-4836; Shuli Man: 0000-0003-4632-3078; Long Ma: 0000-0001-8479-2663

^a^ Co-first author.


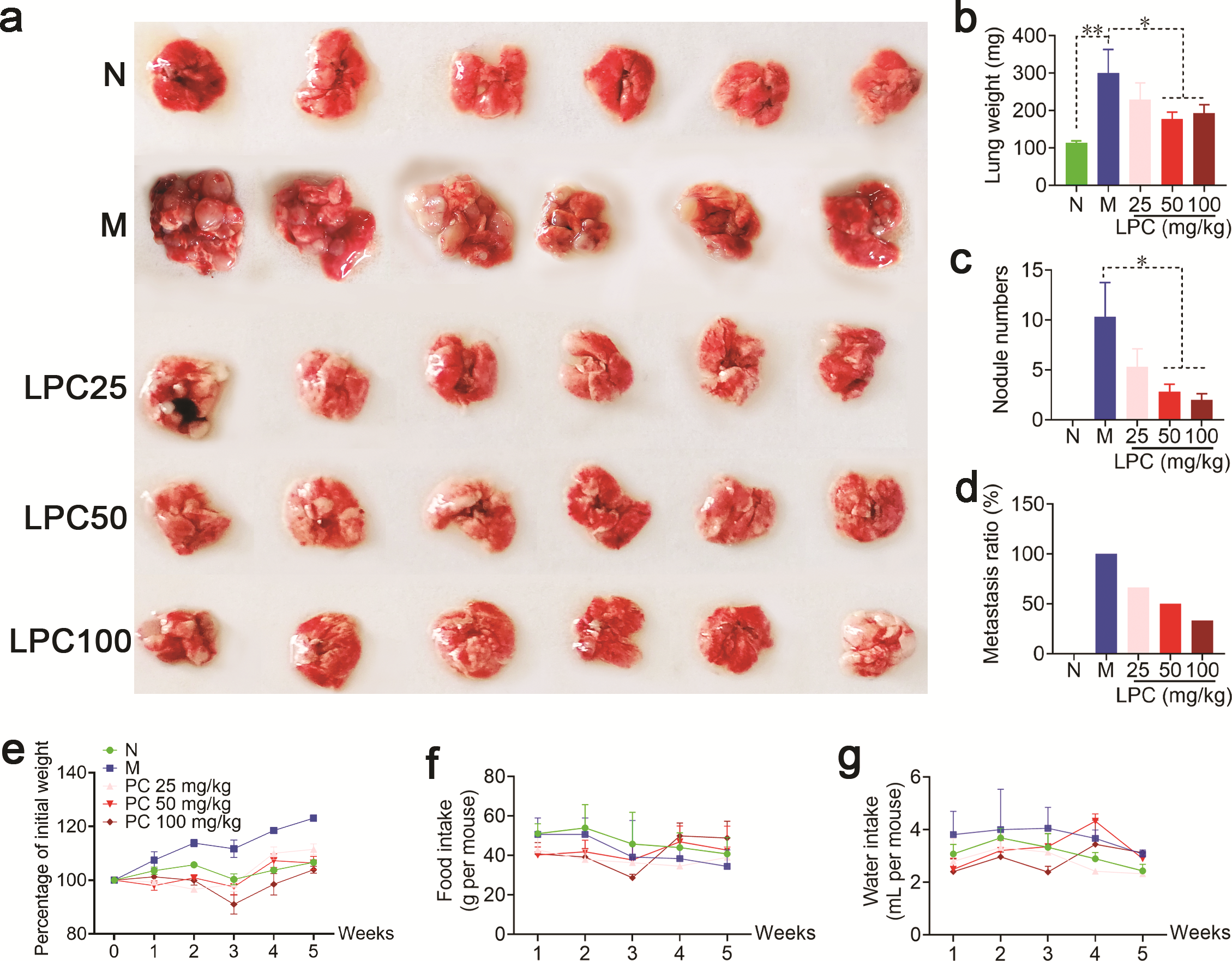


**Fig. S1** **LPC inhibits pulmonary metastasis of CT26 colon cancer.** **a** LPC inhibited CT26 pulmonary metastasis in a dose-dependent manner. **b** Average lung weight of each group. **c** Number of tumor nodules in lungs. **d** Metastasis rate of CT26 cells in each group. **e** Percentage change of body weight. **f-g** Food intake and water intake. Data were presented as means ± SEM, n = 6 mice, **p* < 0.05, ***p* < 0.01.


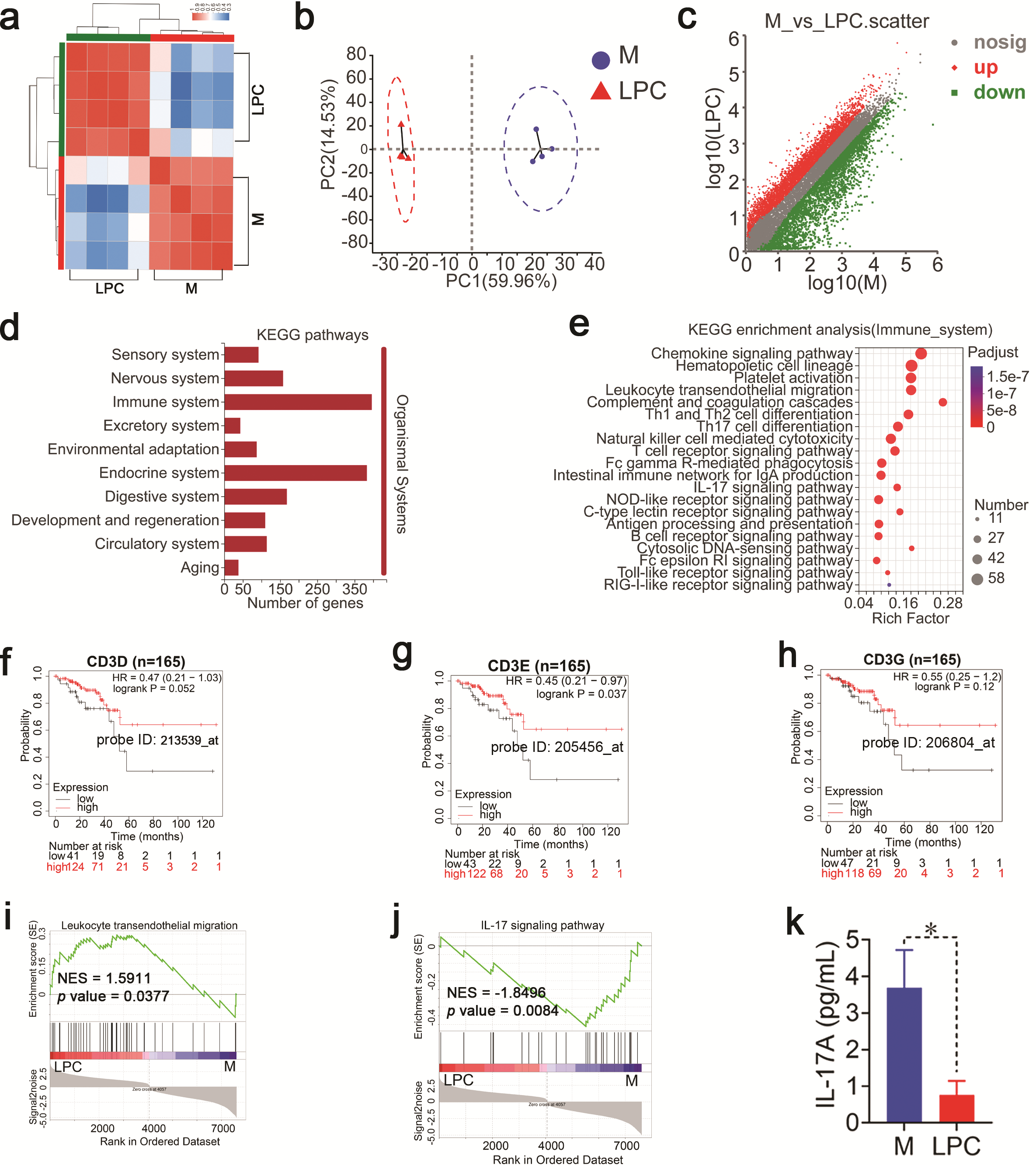


**Fig.** **S2** **LPC regulates T cell immunity in pulmonary metastasic mice**. **a** Correlation analysis between M and LPC group. **b** Two-dimension ordination graph of PCA analysis. **c** Scatter diagram of differentially expressed genes in M and LPC groups. **d** KEGG functional annotation analysis of differential genes. **e** KEGG function enrichment analysis of immune system gene set. **f-h** Kaplan-Meier curves of the relationship between genes expression of CD3D, CD3E, CD3G and survival probability in colorectal cancer patients. **i-j** GSEA based on leukocyte transendothelial migration and IL-17 signaling pathway. **k** The level of serum IL-17A (n = 5 mice). Data were presented as means ± SEM, **p* < 0.05.


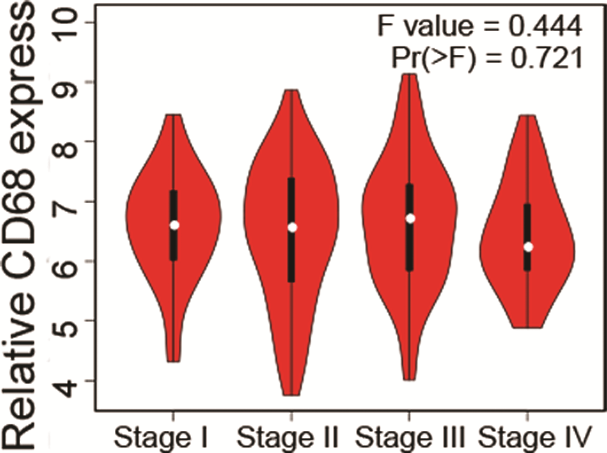


**Fig. S3 Expression profile of CD68 in colorectal cancer patients based on pathological stage.**


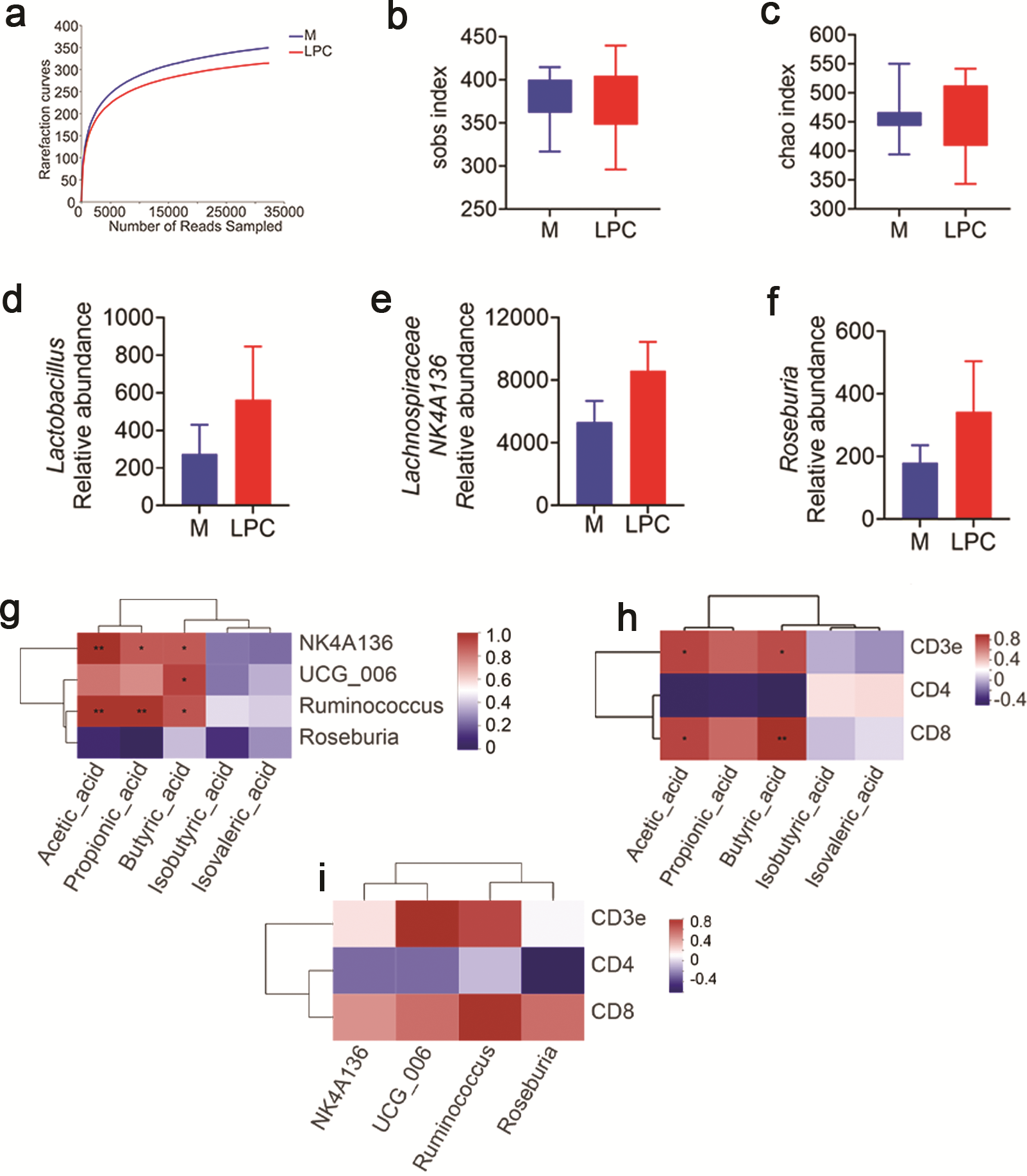


**Fig. S4** **LPC regulates gut microbiota and metabolite in pulmonary metastasic mice.** **a** Rarefaction curves at the OTU level. **b-c** Sobs index and chao index at the OTU level (n = 4 mice). **d-f** Relative abundance of *Lactobacillus*, *Lachnospiraceae_NK4A136* and *Roseburia* (n = 4 mice). **g** Correlation between SCFAs-producing bacteria and SCFAs. **h** Correlation between T cells and SCFAs. **i** Correlation between T cells and SCFAs-producing bacteria. Data were presented as means ± SEM, **p* < 0.05, ***p* < 0.01.


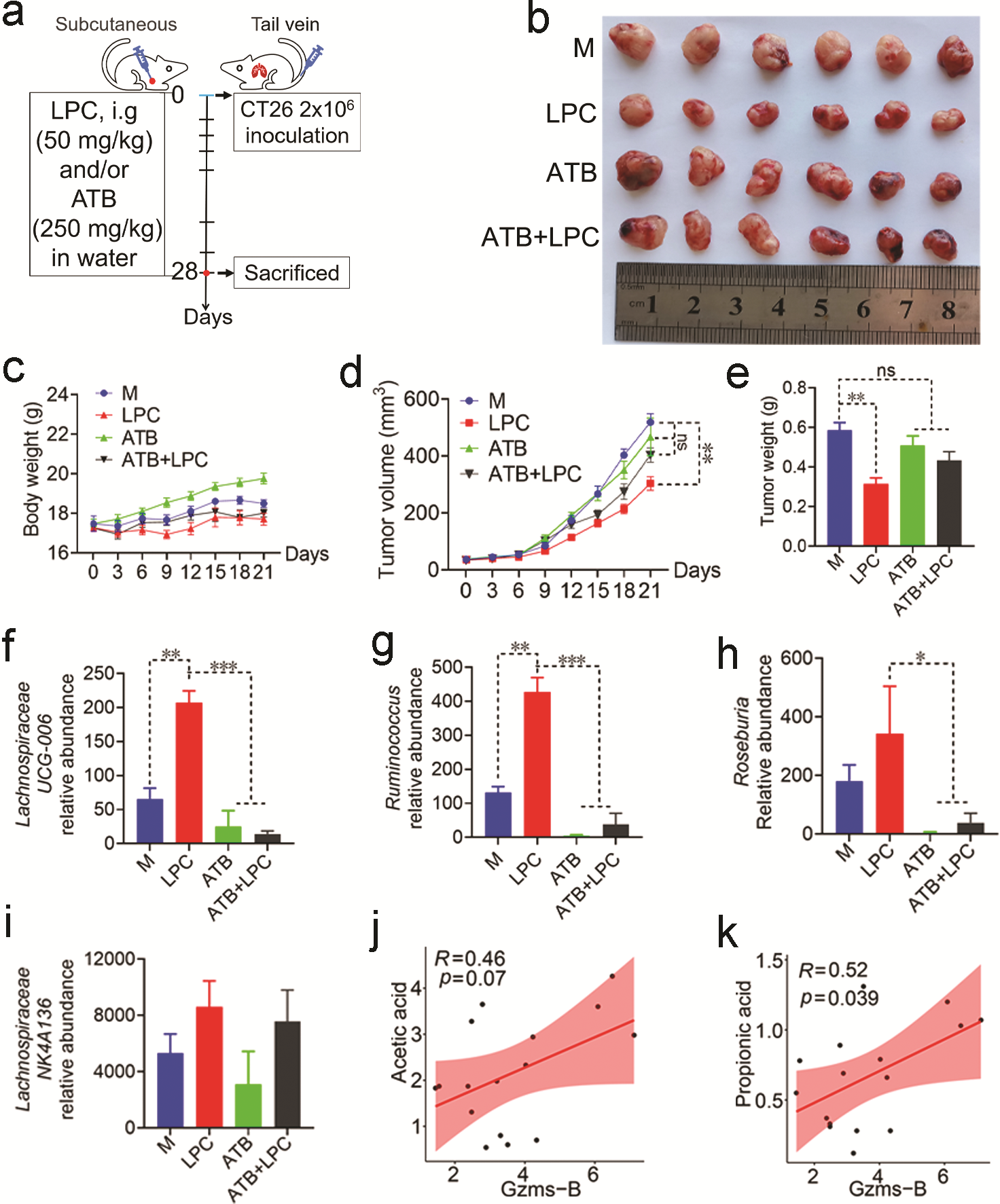


**Fig. S5** **LPC-triggered anti-colon cancer T cell immune response dependents on gut microbiota.** **a** Schematic view of the experimental procedures of LPC+ATB anti-tumor model. **b** Images of CT26 tumors after LPC treatment *in* *vivo*. **c-d** The change of body weight and tumor volumes during the period of experiment. **e** Tumor weight at the end of the experiment (n = 6 mice). **f-i** Relative abundance of *Lachnospiraceae UCG-006*, *Ruminococcus*, *Roseburia* and *Lachnospiraceae NK4A136*. **j-k** Pearson correlation analysis between Gzms-B and acetic acid or propionic acid, R was correlation coefficient. Data were presented as means ± SEM, **p* < 0.05, ***p* < 0.01, ****p* < 0.001.

**Table S1. Primer sequences for RT-PCR**

| **Gene** | **Forward Primer (5’-3’)** | **Reverse Primer (5’-3’)** |
| --- | --- | --- |
| GAPDH | GGAGCGAGATCCCTCCAAAAT | GGCTGTTGTCATACTTCTCATGG |
| CD68 | GGTCAAAGTTACCGGGCAGT | AAGCCCCACTTTAGCTTTACC |
| CD206 | CTCTGTTCAGCTATTGGACGC | CGGAATTTCTGGGATTCAGCTTC |
| CCL2 | TTAAAAACCTGGATCGGAACCAA | GCATTAGCTTCAGATTTACGGGT |
| CSF1r | ATACAGGATAACGATTGACG | GCTCTAGAGATCACATAG |
| ARG1 | AATCCTTCATTCCACCGG | AACGCTTCACGAATTTGCGT |
| TLR4 | ATGGCATGGCTTACACCACC | GAGGCCAATTTTGTCTCCACA |
