# Supplementary figures and images for: Litchi procyanidins inhibit colon cancer proliferation and metastasis by triggering gut-lung axis immunotherapy
Source: Cell Death Dis. 2023 Feb 11;14(2):109. doi: 10.1038/s41419-022-05482-5 (PMC9922286; doi:10.1038/s41419-022-05482-5)

Full and uncropped western blot for Figure 2h


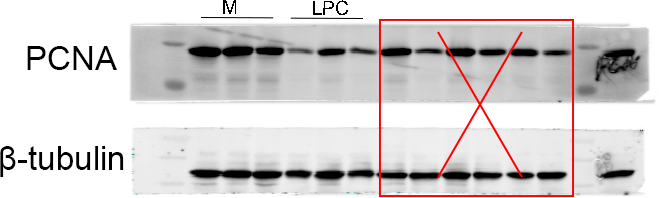


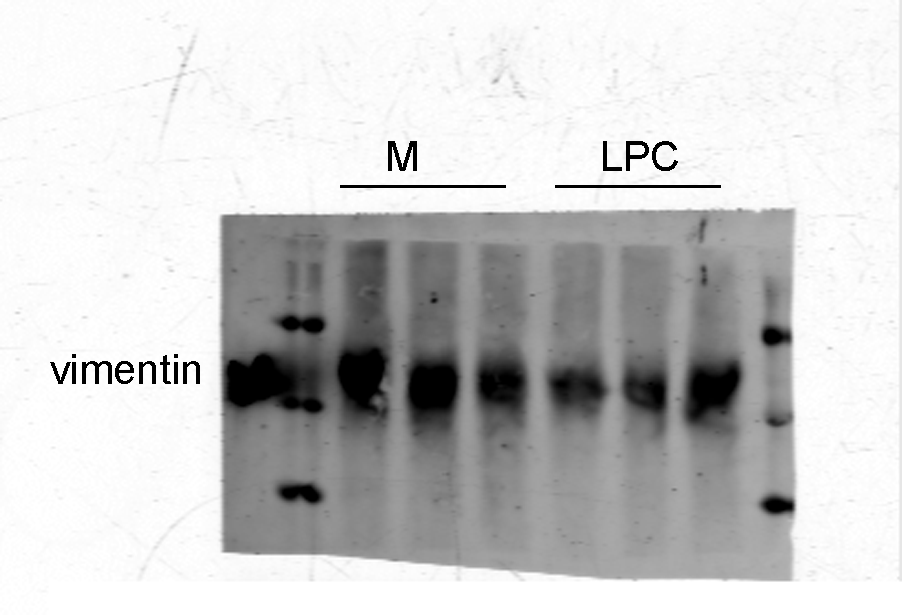


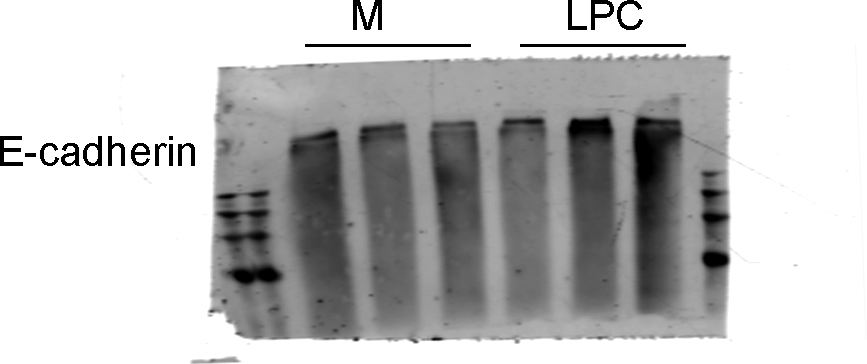

Supplement: Supplementary file 2 — Original Data File [file 41419_2022_5482_MOESM2_ESM.doc]
